# Magnitude and Determinant Factors of Pediatrics Seizures in Pediatrics Emergency Unit at Tikur Anbessa Specialized Hospital, Addis Ababa, Ethiopia, 2020: A Retrospective and Descriptive Study

**DOI:** 10.1155/2023/3967899

**Published:** 2023-07-14

**Authors:** Asaminew Habtamu, Rahel Argaw, Wagari Tuli, Ayalew Moges

**Affiliations:** ^1^Institute of Health Science, School of Nursing, Department of Emergency Medicine and Critical Care Nursing, Jimma University, Jimma, Ethiopia; ^2^School of Medicine and Critical Care, College of Health Science and Medicine, Addis Ababa University, Addis Ababa, Ethiopia

## Abstract

**Background:**

One of the most prevalent medical problems affecting kids is epilepsy, which is the most prevalent chronic neurological condition in kids in both developed and developing nations. The spectrum of diseases that make up childhood epilepsy syndromes ranges from mild to potentially fatal. Children may experience seizures due to a variety of illnesses, such as infection, severe brain injury, and anatomical deformity. It is the foremost visit calculates in neurological and cognitive impedance in children in low-income countries as well as the foremost common reason for pediatric clinic confirmations in children from destitute countries. All things considered, constrained things have been conducted in Ethiopia. Hence, this ponder points to survey the size and related variables of pediatric seizures among children conceded to Tikur Anbessa pediatric emergency.

**Methods:**

By looking through 256 patient charts, an institution-based retrospective cross-sectional analysis was done. Data collection utilized a systematic checklist that had been evaluated beforehand. The EpiData version 4.4.2.2 was used to enter the data, which was then exported for analysis to the SPSS version 25. Binary logistic regression and descriptive statistics were employed. Variables from the bivariate analysis that had a *p*-value of less than 0.25 were carried over to multivariate analysis. The strength and existence of the link were assessed using adjusted odds ratios with a 95% confidence interval and a *p*-value of 0.05, respectively.

**Result:**

Patients' ages ranged from 3.7 to 2.8 years on average. 4.5% of patients had seizures, and 155 of them (60.5%) were men, with a male-to-female ratio of (1.5 : 1). Males and females experienced seizures on average at 1.3 (95% CI: 1.1, 1.5) and 2 (95% CI: 1.6, 2.5) years old, respectively. AOR: 2.68 (95% CI: 1.192, 6.68, *p* = 0.02) and AOR: 2.8 (95% CI: 2.576, 5.302, *p* = 0.04) both demonstrated statistically significant correlations with pediatric seizure.

**Conclusion:**

A vast number of children experienced convulsions, with generalized seizures being the most prevalent form. The chances of a child having a seizure were linked to their family history and where they were born. Therefore, health workers and other people who are involved in healthcare need to work harder on the factors that they have already identified.

## 1. Introduction

A seizure is a malfunctioning neuronal activity that ends in behavioral, sensory, perceptual, or motor activities. Seizures are divided into four primary groups based on the location of commencement [1]. Seizures account for 5% of the worldwide burden of disease, which affects people everywhere, regardless of age, race, financial status, or geography [2]. The World Health Organization study group in 2015, especially in developing nations where seizures can have life-threatening implications, ranks as a top priority for control. More than three million Americans of all ages suffer from seizures. Each year, over 200,000 new instances are reported, and 40–50% of those episodes will return and be labeled as epilepsy [3].

In children, epilepsies are a significant contributor to neurological morbidity. From birth to age 15 years, the typical annual rate of new cases (incidence) of epilepsy is about 5–7 cases per 10,000 children. This means that approximately 5 out of every 1,000 children will have epilepsy in any given year. Some data suggest the prevalence of epilepsies in some child populations may be declining with time; this idea warrants additional research. Males are somewhat more prone compared with females to acquire epilepsy [2, 3].

One of the most prevalent medical problems affecting kids is seizures, which are also the most prevalent chronic neurological condition in kids in both developed and developing nations. The spectrum of diseases that make up childhood epilepsy syndromes ranges from mild to potentially fatal. Children may experience seizures for a variety of reasons, such as infection, severe brain injury, and anatomical deformity [4].

In high-income countries, the youngest age groups have the highest prevalence of pediatric seizures. Neonatal seizures, central nervous system (CNS) infections, and childhood static encephalopathy were the most significant risk factors for newly diagnosed pediatric seizures even though the majority of these patients had no known putative risk factors [5]. Additionally, a Turkish study discovered that neurological impairment, head trauma, a poor Apgar score, and a family history of epilepsy were the top risk factors for childhood seizures [6].

Seizures are among the most frequent causes of hospital hospitalizations for young infants, especially in underdeveloped nations. Regional variety in poor countries has an impact on the typical causes of seizures in a certain location, according to a study The etiological factors that lead to seizures in children are different from those that lead to seizures in adults. Perinatal trauma, CNS disorders, hereditary conditions, and high fever are the main causes of seizures in young children. Malaria, meningitis, and head traumas are CNS disorders that can affect people of any age [4, 5].

Epilepsy is a public health concern in Ethiopia, where the estimated prevalence is 5.2/1000 at-risk individuals, and the yearly incidence is 64 per 100,000 people, with a male prevalence of 5.8% and a female prevalence of 4.6% [8].

Epilepsy and seizures are extremely common in Ethiopia. A high rate of spontaneous remission of epilepsy and/or high mortality due to epilepsy may be used to explain a high incidence of epilepsy in the study area and a prevalence comparable with the rest of the world. Only a small percentage of respondents received Automated Electronic Defibrillator treatment despite community health education on epilepsy, which may be a sign of patient transportation concerns and limits in healthcare facilities [9].

The most prevalent, serious, and undertreated neurological illness in children is epilepsy, which continues to be one of the main causes of impairment in Ethiopia. More than 1 million to 500,000 people are estimated to have epilepsy, with 500,000 of those being projected to have current epilepsy (seizures during the past two years). Many epileptic children go on to have lower education levels as adults, which limits their employment options and increases poverty. Only 5% of the 1 million people who have epilepsy at this time seek medical attention, which means that 95% do not receive any. Ignorance of the causes of epilepsy has been related to unfavorable attitudes, notions, and stigma in the public, at work, and in institutions of higher learning [10]. The purpose of this study was to estimate the proportion of pediatric seizures and associated factors among children hospitalized at Tikur Anbessa Specialty Hospital, to prepare for future interventions to address their source and decrease seizure frequency while sparing families from unnecessary costs.

## 2. Methods

Reviewing 256 patient charts of kids with pediatric seizures admitted at Tikur Anbessa Hospital between December 2016 and December 2019 allowed researchers to conduct an institutionally based retrospective cross-sectional study. A total of 13,921 kids were admitted at the pediatric emergency department overall throughout these three years, 8429 of them were boys and 5492 were girls, and 666 of them were having a pediatric seizure. All children with seizure disorders between the ages of one month and twelve years were included in the study population. Patients under the age of eighteen years old and those whose records were lost from the record office due to consultation, transfer, or any other medical cause were also not included. In addition, data were gathered using a pretested, organized checklist. Afterward, the data were transferred to the SPSS version 25 for analysis after being entered into the EpiData version 4.4.2.2. Binary logistic regression and descriptive statistics were employed. Variables from the bivariate analysis that had a *p*-value of less than 0.25 were carried over to multivariate analysis. The strength and existence of the link were assessed using adjusted odds ratios with a 95% confidence interval and a *p*-value of 0.05, respectively [15].

### 2.1. Sample Size Determination and Sampling Procedure

The stat. calc program of Epi Info version 7.2.5 was used to estimate the necessary sample size using the single population proportion method after taking into account the following factors: 95% confidence level, 50% proportion of pediatric, and 5% degree of freedom. A sample size of 267 is the result. The samples were then chosen afterward using a methodical random sampling procedure. By dividing the study population by the sample size, the sampling interval (*K*), which was established to be three, was calculated. As a result, every third chart was evaluated after the lottery approach chose the first case.

### 2.2. Operational Definitions and Measurements

#### 2.2.1. A Generalized Seizure

It starts somewhere or quickly affects bilaterally spread networks. These networks can be in the subcortical or cortical regions, and they are commonly both.

#### 2.2.2. Focal Seizure

This category encompasses all other focal, unclear, and seizure-like seizures.

#### 2.2.3. Early Onset of Pediatric Seizure

It is the entourage's age at the first unprovoked seizure (onset) was 1 year (SD 2.2 years).

### 2.3. Data Collection Tools and Quality Assurance

A standardized and tested data extraction checklist that was created from patient registration follow-up and was based on prior research was used to gather the data [6, 16–19]. The supervisor examined the data for completeness every day after it was collected. It consists of socio-demographic characteristics, clinical and laboratory investigations, comorbidities, and types of seizures. Training on the basics of the questionnaire, and data collection tool was given to four data collectors (MSc nurse) and one supervisor (MSc nurse) to extract the data from the patient's charts.

### 2.4. Data Processing and Analysis

The EpiData version 4.4.2.2 was used to enter the data, which was then exported to the SPSS version 25 for analysis. The frequency distributions, proportions, and numerical determine were used to describe the data, which were exported to the SPSS version 25 for analysis. Binary logistic regression was used to conduct inferential statistical analysis. Bivariate analysis was performed to ascertain the association of each independent variable with pediatric seizures, and variables with a *p*-value of 0.25 were candidates for a multivariable model. Multivariable analysis was run for confounder adjustment after conducting a model fitness test using Hosmer and Lemeshow test. Then, an adjusted odds ratio with a 95% confidence interval was used to assess the presence and strength of the association, whereas statistical significance was declared at the *p*-value of <0.05.

### 2.5. Ethical Declaration

The study received institutional review board (IRB) approval from Tikur Anbessa. The clinical director of the pediatric emergency directorate was then given an official letter of collaboration from the research directorate. After receiving approval from the clinical director and record room officials, data gathering carried out. The consent of the study participants' and their families was waived because it was a retrospective study. The study was conducted following the Declaration of Helsinki and its ethical guidelines. By using an anonymous data-gathering process, the study's confidentiality was preserved at all points.

## 3. Results

### 3.1. Socio-Demographic Characteristics

A 96% response rate was achieved when families were contacted to discuss any missing paperwork from the 256 child charts that were examined. 4.5% of all children experienced seizures, and a male predominance was found with a male-to-female ratio of 1.5 : 1. The patients' average ages ranged from 3.7 to 4.5 years, with a standard deviation of 2.8 years (95% CI: 3.1, 4.5). Pediatric seizures were diagnosed in 154 (60.2%) of the children under the age of 1 year, with a mean age of 1.6 years (95% CI: 1.4, 1.8), STD, 1.7. Males experienced seizures at an average age of 1.3 years (95% CI: 1.07, 1.47) and girls at an average age of 2.1 years (95% CI: 1.6, 2.5), respectively. Most of the patients were from Addis Ababa (Table 1).

### 3.2. Clinical Parameter

This study found that generalized tonic-clonic seizures, which happened in 205 cases (80.1%) were the most frequent type of seizure, followed by partial (focal) seizures, which occurred in 51 instances (19.9%). There were between one and twelve seizures per patient. According to the findings, 127 individuals (49%) had at least four seizures, the majority of which lasted under a minute. Electroencephalograms were performed on 160 kids, and 158 (68.1%) of them exhibited abnormal tracings (Table 2).

**Figure 1:**
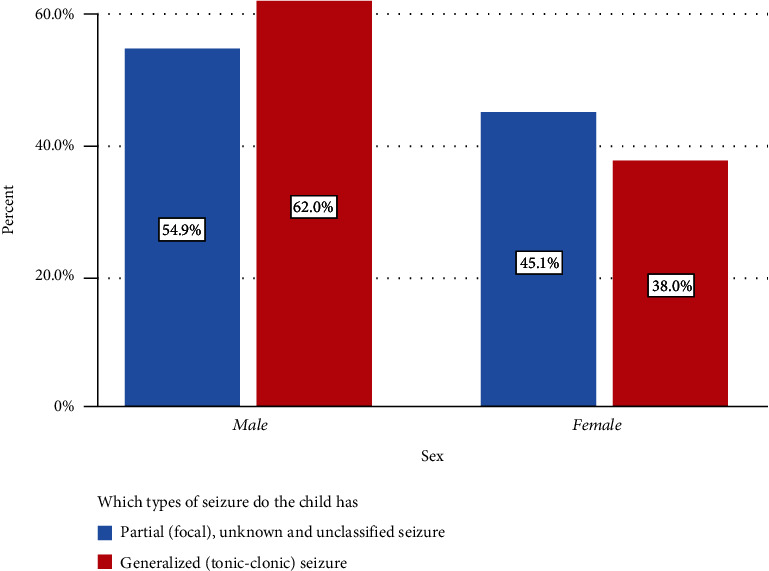
The most common types of a pediatric seizure based on the sex of child at emergency department of Tikur Anbessa Specialized Hospital, 2020.

A generalized (tonic-clonic) seizure is common in men, accounting for 49.6% of cases, whereas it is uncommon in women, accounting for 30.4% of cases when we categorized seizures according to genders (Figure 1).

### 3.3. Description of Presenting Symptoms

The study's findings also showed that among patients with admitted seizures, 235 (91.8%), 120 (46.9%), 68 (26.8%), and 37 (14.5%) had the four most common clinical complaints: abnormal body movement, fever, vomiting, and headache (Table 3).

### 3.4. Description of the Medical Condition among Study Participants

This study found that 198 patients out of 138 suffered a head injury (77.3%), and the majority of them (96.9%) had no history of stroke. The youngsters had brain tumors and vascular abnormalities in 6.3% and 7.8% of them, respectively, according to the findings. According to the study, only 10.5% of children had a family history of seizures, and the vast majority of them (80.9%) were born at term. This finding revealed that 79.3%, 19.9%, and 8% of children were born via natural birth via the vagina, cesarean section, and facilitated vaginal delivery, respectively (Table 4).

### 3.5. Pediatric Seizure and Determinant Factors

In the bivariate analysis, factors with a *p*-value of less than 0.25 were identified as candidate variables for multivariable analysis, including sex, place of residence, brain injury, place of the birth, parental history of seizures, cerebral infection, developmental history of neonatal distress, and immunization history. Following model fitness and other assumption tests, multivariable analysis was performed with these variables included for confounder adjustment. Last but not least, a 95% confidence level revealed a statistically significant link between the existence of generalized pediatric seizures and the place of birth and seizure family history. According to the study's findings, patients with a family history of seizures were 2.7 times more likely than those without one to experience generalized juvenile seizures [(AOR = 2.7; 95% CI: 1.2, 6.7), *p* = 0.02]. When patients were delivered at home as opposed to a hospital or health facility, the odds of generalized pediatric seizures were 2.8 times greater [(AOR = 2.8; 95% CI: 2.6, 6.5, 47.3), *p* = 0.007] (Table 5).

## 4. Discussion

Younger kids are more likely to experience seizures than older kids are, and more boys experience them than girls. This is seen in the current study, which showed that younger age groups (0 years; 7–12 years) had a higher prevalence of seizures and that men were more likely than women to have seizures (male/female = 1.5 : 1). According to this finding, 4.5% of all children admitted within those three years were pediatric patients with seizures. This outcome is remarkably similar to studies conducted in Nepal, southern Brazil, and the UK [5, 8, 15]. Men may also take more risks than women, which raises the possibility that they will sustain brain damage, explaining why men outnumber women. However, a study conducted in Kenya and Egypt found that the prevalence of pediatric seizures was 8% and 11%, respectively, which was incongruent with the current finding [4, 14]. The inclusion of neonates experiencing seizures in their study may be the cause of this inconsistency. The maximum age group in their study was up to 18 years, however, in our study, it was below 12 years. In addition, newborns or children under the age of 1 year having seizures were not included in our study.

Children's median age of start was one year, and their median seizure time was two minutes. This is in line with a study conducted in a hospital in our nation that found the median age of onset was one year and the median duration was two minutes [23]. According to a study conducted in Ghana and Kenya, the median and mean age at which seizures occurred were 8.0 and 2 years, respectively, whereas the median length of seizures was 10.2 minutes [14, 22]. This variance could be attributed to several demographic variables, including the study participants' age groups, which ranged from 1 month to 18 years old in their case, as well as the duration difference.

In accordance with this study, generalized tonic-clonic seizures, also known as tonic—are the most common type of child seizures, with men accounting for 49.6% of all generalized (tonic-clonic) seizures. This finding is congruent with the findings of a study conducted in the United Kingdom, Nepal, Ghana, and Ethiopia, which revealed that generalized tonic-clonic seizures were the most common and that men were more likely to experience them when compared with women [5, 15, 22, 23].

With a *p*-value of less than 0.25 in the bivariate analysis, variables like sex, residency, head injury, place of delivery, family history of seizures, CNS infection, delay in the developmental history of newborn distress, and immunization history were identified as candidate variables for multivariable analysis. Multivariable analysis was conducted after model fitness and other assumption tests, using these variables for confounder adjustment. Finally, it was discovered that there was a statistically significant link between the presence of generalized pediatric seizures and the place of birth and family history of seizures at a 95% confidence level.

Patients with a family history of seizures were found to be 2.7 times more likely to have generalized pediatric seizures [(AOR = 2.7; 95% CI: 1.2, 6.7), *p* = 0.02] than those with no family history of seizures. It was consistent with research conducted in our country, which found that 22% had a family history of seizures and generalized seizures [13]. It was also consistent with a study conducted in Nepal and Kenya, which found that people with familial seizures are more likely to have generalized seizures 2.75 (95% CI: 1.2, 6.0, *p* = 0.03) [8, 13]. It was also consistent with a Turkish study that found that familial seizure increases the likelihood of getting seizures by 10.9 times [19].

When patients were delivered at home as opposed to a hospital or health facility, the likelihood that they would experience generalized pediatric seizures was 2.8 times higher [(AOR = 2.8; 95% CI: 26.65, 47.3), *p* = 0.007]. These results were consistent with a study conducted in Ghana, which revealed that having a child at home can increase the risk of having a child have a seizure [(AOR = 2.7; 95% CI: 1.3, 7.2, *p* = 0.01)] [20, 22]. Even while our study did not demonstrate any significance, the majority of previous studies revealed that head injury is more frequently linked to seizures. This might be because the study group's age range varied from the general population. The absence of the right classification in the archive as well as the inaccurate recording of clinical examination descriptions and outcomes in the records were some of the challenges this study faced. Because the study is cross-sectional, the causal relationship between the dependent and independent variables in the analysis may not be shown. A community-based study is the best sort of research to uncover risk variables. We excluded children with seizures from the outpatient clinic and infants in the neonatal intensive care unit (NICU).

## 5. Conclusion

Nearly 5% of the children who entered the pediatric emergency room suffered seizures. Additionally, it was found that the prevalence of seizures was higher at younger ages. The most frequent type of seizures was generalized tonic-clonic, and most cases began in newborns under a year old. The only two variables in this study that were significantly linked with a child having a seizure were family history and whether the child was born at home. At some point, it must argue for the necessity of providing proper prenatal and postpartum care.

## Figures and Tables

**Table 1:** Socio-demographic characteristics of pediatric seizures at emergency departments of Tikur Anbessa Specialized Hospital, Addis Ababa, Ethiopia, 2016–2019 (*n* = 256).

Parameters	Category	Frequency	Relative frequency (%)	Sample statistics
Age in years	<1	52	20	Mean = 3.73, standard deviation = 2.78
	1–3	77	30.02	
	3–6	93	36.2	
	7–12	34	13.2	
	Total	256		
Sex	Female	101	60.5	
	Male	155	39.5	
Residency	Addis Ababa	174	68.0	
	Oromia	49	19.1	
	Amhara	15	5.9	
	Others	18	7.0	
Occupation family	Salaried worker	83	32.4	
	Trader	98	38.3	
	Farmer	72	28.1	
	Others	3	1.2	
Drop out of school	Yes	56	21.9	
	No	200	78.1	

**Table 2:** Clinical parameter of pediatric seizure at emergency departments of Tikur Anbessa Specialized Hospital, Addis Ababa, Ethiopia, 2016−2019.

Parameters	Category	Frequency	Relative frequency (%)	Sample statistics
Pediatric seizure	Generalized (tonic-clonic) seizure	189	73.8	
	Partial seizure	41	16	
	Unclassified	14	5.5	
	Unknown	12	4.7	
Age at onset	Below one year	154	60.2	Mean = 1.6, median = 1, range = 9
	Greater than one year	102	39.8	
Nutritional status	Normal (BMI >20, −2 < *Z*)	74	28.9	
	Underweight(BMI <10, −2 < *Z* > −3)	125	49.1	
	Wasted (BMI Z < −3)	57	22.2	
Length of episode (minutes)	Below 1	56	21.9	Mean = 2.37 minute, median = 2 minute, IQR = 1.2 minute
	1−3 minute	92	35.2	
	4−5	70	27.4	
	6−30	33	12.9	
	≥30	5	2.0	
Was the EEG done	Yes	160	62.5	
	No	96	37.5	
If yes what was the result	Normal	2	0.8	
	Abnormal	158	61.7	
Event frequency	Once per day	9	3.5	Std = 1.692739, mean = 3.22, standard deviation = 0.894
	Twice per day	52	20.3	
	Three times per day	68	26.8	
	≥4	127	49.6	

**Table 3:** Clinical sign and symptoms at admission of the pediatrics seizure at the emergency department of Tikur Anbessa Specialized Hospital, Addis Ababa, Ethiopia, 2016–2019.

Parameters	Parameter category	Frequency	Percentage (%)
Fever	Yes	120	46.9
	No	136	53.1
Headache	Yes	37	14.5
	No	219	85.5
Vomiting	Yes	68	26.8
	No	188	73.4
Aphasia	Yes	21	8.6
	No	235	91.8
Altered mental status	Yes	71	27.5
	No	185	72.3
Abnormal body movement	Yes	235	91.8
	No	22	8.2

**Table 4:** Medical and perinatal conditions of the pediatric seizure at the emergency department of Tikur Anbessa Specialized Hospital, Addis Ababa, Ethiopia, 2016–2019.

Parameter	Parameter category	Types of pediatric seizures		Total
		Focal	Generalized	
Newborn distress	Yes	7	44	51
	No	44	161	205
Place of delivery	Health center	14	88	154
	Home	37	117	55
Electrocardiogram	Yes	32	128	160
	No	19	77	98
Head trauma	Yes	42	156	198
	No	9	49	58
Stroke	Yes	3	5	8
	No	46	198	244
Neurodegenerative	Yes	3	6	9
	No	46	198	244
Family history of seizure	Yes	14	164	53
	No	37	196	201
Immunization status	Yes	49	196	245
	No	2	7	9

**Table 5:** Association of factors of pediatric seizures by using logistic regression in pediatrics emergency among 1 month to 12 years of age children admitted at the Tikur Anbessa Specialized Hospital pediatric emergency unit.

Parameter	Categories	Pediatric seizure		COR (95% CI)	AOR (95% CI)	*p*-Value
		Generalized pediatric seizure	Focal pediatric seizure			
Sex	Male	127	28	1.337 (1.01, 2.485)	1.611 (1.017, 2.364)	0.32
	Female	78	23	1	1	
Residency	Addis Ababa	144	30	23.840 (1.399, 10.58)	1.936 (1.124, 3.092)	0.96
	Oromia	39	10	3.12 (1.702, 9.58)	4.43 (1.305, 6.836)	
	Amhara	12	3	3.2 (.66, 15.029)	0.236 (0.0125, 1.25)	
	Others	10	8	1	1	
Head trauma	Yes	155	42	1.466 (1.781, 3.224)	1.465 (1.05, 2.836)	0.22
	No	49	9	1	1	
Fever	Yes	108	20	1.7260 (1.050, 3.226)∗	1.82 (1.02, 2.256)∗∗	0.51
	No	97	31	1	1	
Hypoxic brain ischemia	Yes	39	14	1.5910 (1.042, 1977)∗	2.43. (0.624, 5.996)∗∗	0.14
	No	164	37	1	1	
Time of birth	Premature	34	11	0.2833 (0.748, 8.227)	17.292 (−0.876, 0.966)	0.25
	Term	159	29	5.026 (2.305, 12.836)	0.219 (0.011, 4.37)	
	Post term	12	11	1	1	
Place of delivery	Health center/hospital	88	14	1.988 (1.013, 3.901)	2.8 (2.076, 5.302)∗∗	0.04
	Home	117	37	1	1	
Family with A seizure	Yes	68	23	1.743 (1.071, 3.963)	2.68 (1.193, 6.652)	0.02
	No	134	26	1	1	
Cns infection	Yes	73	11	2.022 (1.756, 1.897)	0.24 (0.876, 1.59)	2.399
	No	128	39	1	1	
Delays developmental	Yes	137	31	1.32 (0.992, 0.029)	0.861 (0.12, 1.59)	0.885
	No	68	20	1	1	
History of Newborn's distress	Yes	44	7	0.582 (0.2429, 1.9630)∗	0.341 (−0.761, 0.856)∗∗	0.483
	No	161	44	1	1	
Immunization history	Yes	196	49	1.143 (.247, 5.029)	0.772 (0.01, 1.06)	0.389
	No	7	2	1	1	

COR: crude odd ratio; CI: confidence interval; AOR: adjusted odds ratio; CNS: central nervous system.

∗, ∗∗Used to separate crude ratio from adjusted ration during analysis.

## Data Availability

Data supporting this research article are available from the corresponding author or first author on reasonable request.

## Abbreviations

AAP: America Academic of Pediatrics
CNS: Central nervous system
dRPC: Development research and projects center
CT: Computed tomography
EEGs: Electroencephalographs
FS: Febrile seizure
GTCS: Generalized tonic-clonic seizures
ICH: Intracranial hemorrhage
MRI: Magnetic resonance imaging
NCC: Neurocysticercosis
OR: Odds ratio, countries with few resources
SAH: Subarachnoid hemorrhage, Epileptics with status
SMR: Standard mortality rate
WHO: World Health Organization
TASH: Tikur Anbessa Specialized Hospital.

## Additional Points

*Study Limitations.* Because babies in the region where our inquiry was done should get critical care in a NICU, children under the age of 1 year were omitted from our study. Another problem is the failure to assess the patient's response to emergency therapy. Because it was only conducted in one area, this study may not be as generalizable as those that were conducted in multiple sites.
